# Targeting Apoptosis-Resistant Proliferation: Imatinib-Based Combinations Induce Durable Cytostatic Arrest in 3D Endometrial Cancer Spheroids

**DOI:** 10.3390/biomedicines14040906

**Published:** 2026-04-16

**Authors:** Berna Yıldırım, Burcu Biltekin, Mete Hakan Karalök, Ayhan Bilir

**Affiliations:** 1 Department of Histology and Embryology, Faculty of Medicine, İstanbul Atlas University, 34403 İstanbul, Turkey; 2 Department of Obstetrics and Gynecology, Faculty of Medicine, İstanbul Atlas University, 34403 İstanbul, Turkey

**Keywords:** endometrial cancer, 3D tumor spheroids, imatinib, lithium chloride, medroxyprogesterone acetate, cytostatic arrest, apoptosis resistance

## Abstract

**Background/Objectives:** Endometrial cancer frequently develops resistance to apoptosis-based therapies, highlighting the need for alternative strategies that control tumor growth independently of cell death induction. Three-dimensional (3D) tumor models more accurately recapitulate tumor architecture, cellular interactions, and treatment resistance compared to conventional two-dimensional (2D) cultures. This study aimed to investigate whether imatinib-based combination treatments can enforce sustained cytostatic responses in a 3D endometrial cancer model. **Methods:** Ishikawa spheroids were treated with imatinib alone or in combination with lithium chloride or medroxyprogesterone acetate. Proliferation was assessed by bromodeoxyuridine incorporation, cell cycle distribution by flow cytometry, and apoptosis by Annexin V/propidium iodide staining over 96 h. **Results:** Imatinib monotherapy produced modest antiproliferative effects, whereas combination treatments resulted in sustained suppression of DNA synthesis, increased G_0_/G_1_ accumulation, and reduced S-phase entry. Despite strong growth inhibition, apoptotic fractions remained low across all groups. **Conclusions:** Imatinib-based combinations suppress 3D endometrial cancer growth predominantly through sustained cell cycle arrest rather than apoptosis induction. Targeting apoptosis-resistant proliferation through cytostatic mechanisms may represent a complementary therapeutic strategy for hormone-responsive endometrial cancer and warrants further translational evaluation.

## 1. Introduction

Endometrial cancer is the most common gynecologic malignancy in developed countries, and its incidence continues to rise steadily [1]. Although early-stage endometrioid tumors are often effectively managed with surgery and adjuvant therapy, advanced, recurrent, and high-grade subtypes remain associated with limited therapeutic options and poor clinical outcomes [2,3,4]. In these settings, resistance to apoptosis-inducing treatments represents a major clinical challenge. While molecularly targeted therapies have improved survival in several solid tumors, progress in endometrial cancer has been comparatively modest, with durable responses largely restricted to hormone receptor-positive disease and mismatch repair-deficient tumors [5,6]. Consequently, there is a pressing need for alternative strategies that regulate tumor growth independently of classical apoptotic mechanisms and may complement existing systemic therapies.

In this context, therapeutic approaches that enforce sustained proliferative arrest rather than acute cytotoxicity have gained increasing interest. Non-apoptotic cytostatic control may represent a clinically relevant strategy, particularly in tumors exhibiting resistance to apoptosis or adaptive survival signaling. Three-dimensional (3D) tumor models offer a translationally relevant experimental platform for studying such responses, as they more closely recapitulate tumor architecture, cellular heterogeneity, and therapy resistance observed in vivo compared with conventional monolayer cultures [7]. Indeed, 3D culture systems have been shown to better predict therapeutic responses and drug resistance patterns across multiple cancer types, thereby improving the physiological relevance of preclinical findings [8,9,10,11,12].

Imatinib mesylate is a clinically established tyrosine kinase inhibitor targeting BCR-ABL, c-KIT (CD117), and platelet-derived growth factor receptors (PDGFRs) [13]. Although highly effective in hematologic malignancies and gastrointestinal stromal tumors, imatinib monotherapy has shown limited efficacy in unselected patients with advanced endometrial carcinoma [14]. This suggests that combinatorial strategies may be required to enhance its biological impact in this disease context. Preclinical data indicate that imatinib may preferentially affect proliferative tumor subpopulations, potentially modulating growth dynamics rather than inducing overt cytotoxicity [15]. In line with this, several studies have suggested that tyrosine kinase inhibitors may exert cytostatic rather than cytotoxic effects in solid tumor models, particularly under conditions that mimic the tumor microenvironment [16].

Lithium chloride (LiCl), a long-standing psychiatric medication, has been investigated in oncology due to its modulation of glycogen synthase kinase-3β (GSK-3β) and its effects on cell cycle regulation and autophagy-associated pathways [17,18,19,20]. Lithium has been reported to induce growth arrest and modulate survival pathways across multiple cancer types, often without triggering classical apoptosis [17,19]. Similarly, medroxyprogesterone acetate (MPA) remains clinically relevant in hormone-responsive endometrial disease, where it promotes differentiation and G_0_/G_1_ cell cycle arrest [21,22,23,24]. Both agents have demonstrated cytostatic activity in preclinical 3D endometrial cancer models [21,25], suggesting that they may reinforce growth suppression when combined with targeted therapies.

Preliminary studies using 3D Ishikawa spheroids have suggested that combining imatinib with LiCl or MPA enhances growth inhibition compared with single-agent treatment [26,27]. However, these effects have primarily been evaluated in limited experimental settings, and the extent to which such combinations induce sustained, non-apoptotic growth arrest in a structured tumor context remains unclear. Therefore, the present study aimed to evaluate whether imatinib-based combinations can induce sustained, non-apoptotic cytostatic arrest in a 3D endometrial cancer model. By integrating proliferation assays, cell cycle analysis, and apoptosis profiling over prolonged exposure periods, we demonstrate that these combinations enforce durable G_0_/G_1_ arrest without significant apoptosis induction. These findings support a translational framework in which cytostatic enforcement may complement existing therapeutic strategies for apoptosis-resistant endometrial cancer.

## 2. Materials and Methods

### 2.1. Cell Line and Culture Conditions

The human endometrial adenocarcinoma cell line Ishikawa (Sigma-Aldrich, St. Louis, MO, USA) was used as an in vitro model of well-differentiated, estrogen receptor-positive type I endometrial carcinoma. Cells were maintained in Dulbecco’s Modified Eagle Medium (DMEM; Gibco, Grand Island, NY, USA) supplemented with 10% fetal bovine serum (FBS), 100 U/mL penicillin, and 100 µg/mL streptomycin. Cultures were incubated at 37 °C in a humidified atmosphere containing 5% CO_2_. Cells were routinely passaged at 70–80% confluence using trypsin–EDTA (0.25% trypsin–EDTA, 3–5 min at 37 °C), and low-passage cultures were used for all experiments to ensure phenotypic stability.

### 2.2. Three-Dimensional (3D) Spheroid Culture

For three-dimensional culture, Ishikawa cells were seeded into ultra-low attachment (ULA) 96-well plates (Corning, Corning, NY, USA) to allow spontaneous spheroid formation. Single-cell suspensions were prepared from semi-confluent monolayers, and 5 × 10^3^ viable cells were plated per well in 200 µL of complete medium. Plates were incubated under standard culture conditions, and compact multicellular spheroids formed within 48 h. Spheroid formation and structural integrity were verified using inverted light microscopy (Zeiss, Oberkochen, Germany) prior to drug treatment. Only uniformly shaped and compact spheroids were included in subsequent analyses. Representative images of spheroid formation were acquired before treatment to confirm structural integrity, and morphological changes during treatment were monitored by light microscopy. Although spheroids were selected based on uniform morphology, quantitative size measurements were not performed, representing a potential source of variability in drug response. Representative images are provided in Supplementary Figure S1.

### 2.3. Drug Treatments

Imatinib mesylate (Gleevec, Basel, Switzerland), lithium chloride (LiCl, St. Louis, MO, USA), and medroxyprogesterone acetate (MPA, St. Louis, MO, USA) were used for experimental treatments. Imatinib and LiCl stock solutions were prepared in sterile distilled water, while MPA was dissolved in dimethyl sulfoxide (DMSO). Working solutions were freshly prepared in complete culture medium immediately prior to use.

Based on previous in vitro studies demonstrating cytostatic effects without overt cytotoxicity in Ishikawa spheroids, the following final concentrations were applied: imatinib (50 µM), LiCl (10 mM), and MPA (200 µM) [26]. These concentrations are within ranges commonly used in preclinical cancer research; however, they may not directly reflect clinically achievable plasma levels and should be interpreted within an experimental context. Control spheroids received equivalent volumes of vehicle (0.1% DMSO). Drug treatments were initiated 48 h after spheroid seeding, once stable 3D structures had formed, and this time point was designated as 0 h. Experimental groups included untreated controls, single-agent treatments, and combination treatments (imatinib + LiCl or imatinib + MPA). Triple combination treatments were not included to allow a clear interpretation of individual drug interactions.

### 2.4. BrdU Proliferation Assay

Cell proliferation within 3D spheroids was assessed using a colorimetric bromodeoxyuridine (BrdU) incorporation assay (Roche Cell Proliferation ELISA, BrdU, Basel, Switzerland). At 24, 48, 72, and 96 h following drug treatment, BrdU labeling solution was added to the culture medium at a final concentration of 10 µM for the final 2 h of incubation.

Following labeling, spheroids were collected and enzymatically dissociated into single-cell suspensions using trypsin–EDTA for 3–5 min at 37 °C with gentle mechanical trituration. Cells were fixed, and incorporated BrdU was detected using a peroxidase-conjugated anti-BrdU antibody according to the manufacturer’s instructions. Absorbance was measured at 370 nm with a reference wavelength of 492 nm using a microplate reader. BrdU incorporation was expressed as a percentage relative to untreated control spheroids. Each condition was analyzed in six technical replicates (*n* = 3 independent experiments).

### 2.5. Cell Cycle Analysis

Cell cycle distribution was analyzed by flow cytometry following propidium iodide (PI) staining. At 24, 48, 72, and 96 h after treatment, spheroids were harvested and dissociated into single-cell suspensions as described above. Cells were washed with phosphate-buffered saline (PBS) and fixed in 70% cold ethanol at −20 °C for a minimum of 2 h.

After fixation, cells were washed, treated with RNase A (50 µg/mL, 30 min at 37 °C), and stained with PI (50 µg/mL) in PBS containing 0.1% Triton X-100. DNA content was analyzed using a BD FACSCanto II flow cytometer (BD Biosciences, San Jose, CA, USA), and at least 20,000 events were acquired per sample. Doublets were excluded by pulse-geometry gating, and cell cycle phases (G_0_/G_1_, S, and G_2_/M) were quantified using FlowJo v10 software. All analyses were performed in triplicate.

### 2.6. Annexin V-FITC/PI Apoptosis Assay

Apoptotic and necrotic cell populations were evaluated using Annexin V-FITC and propidium iodide (PI) dual staining. At 24, 48, 72, and 96 h following treatment, spheroids were collected and dissociated into single-cell suspensions. Cells were washed with cold PBS and resuspended in binding buffer at a concentration of approximately 1 × 10^6^ cells/mL.

Annexin V-FITC (5 µL) and PI (5 µL) were added to 100 µL of cell suspension and incubated for 15 min at room temperature in the dark. Following the addition of binding buffer, samples were immediately analyzed using a BD FACSCanto II flow cytometer. A minimum of 10,000 events per sample was acquired. Cells were classified as viable, early apoptotic, or late apoptotic/necrotic based on Annexin V and PI staining patterns. Data were analyzed using FlowJo software. Experiments were performed in three independent biological replicates.

### 2.7. Statistical Analysis

All quantitative data are presented as mean ± standard deviation (SD) from at least three independent experiments. Statistical analyses were performed using GraphPad Prism version 9. BrdU proliferation data were analyzed using two-way analysis of variance (ANOVA) with treatment and time as factors, followed by Dunnett’s post hoc test for multiple comparisons. Cell cycle distribution and apoptosis data were evaluated descriptively without formal statistical comparison due to the experimental design. A *p*-value < 0.05 was considered statistically significant where applicable. Data distribution was assessed using the Shapiro–Wilk test prior to parametric analysis.

### 2.8. Use of Generative Artificial Intelligence

Generative artificial intelligence (GenAI) tools were not used in the design of the study, data collection, data analysis, data interpretation, or preparation of the manuscript.

### 2.9. Ethical Approval

This study did not involve human participants or animal experiments; therefore, ethical approval was not required.

### 2.10. Data Availability Statement

The data supporting the findings of this study are available within the article. Additional data are available from the corresponding author upon reasonable request.

## 3. Results

### 3.1. Imatinib-Based Combinations Suppress Proliferation in Ishikawa 3D Spheroids in a Sustained, Time-Dependent Manner

**Figure 1:**
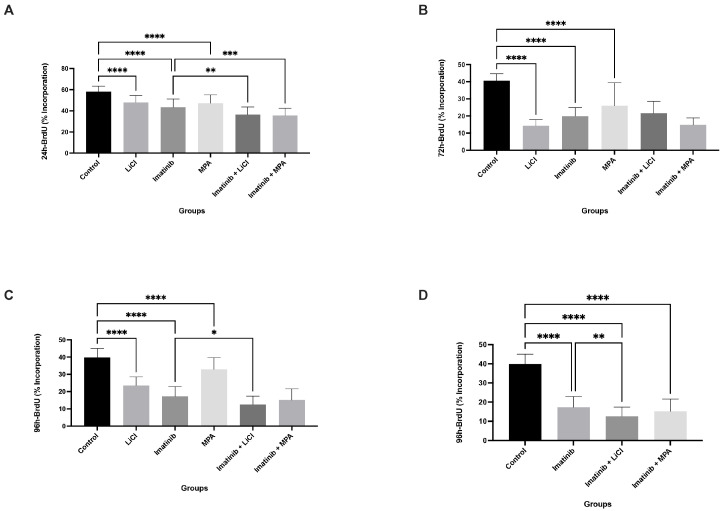
Imatinib-based combinations suppress DNA synthesis in 3D Ishikawa spheroids in a time-dependent manner: (**A**–**D**) BrdU incorporation at 24, 48, 72, and 96 h following treatment with imatinib, LiCl, MPA, and their combinations. (**D**) Focused comparison between imatinib monotherapy and combination treatments at 96 h, excluding single-agent LiCl and MPA groups to highlight combination-specific effects. Data are presented as mean ± SD from three independent experiments (*n* = 3). * *p* < 0.05, ** *p* < 0.01, *** *p* < 0.001, **** *p* < 0.0001 vs. control.

The effects of imatinib, LiCl, and MPA, administered alone or in combination, on proliferative activity were evaluated in 3D Ishikawa spheroids over a 96 h period using BrdU incorporation as an index of DNA synthesis. As shown in Figure 1A–C, untreated control spheroids exhibited sustained proliferative activity across all time points. Representative spheroid images, including Trypan Blue staining, are provided in Supplementary Figure S1 for illustrative assessment of structural integrity and cell viability.

Imatinib monotherapy induced a moderate reduction in BrdU incorporation that became more pronounced over time. While only partial suppression was observed at 24 h (Figure 1A), BrdU incorporation decreased to approximately 15–25% at later time points (Figure 1B,C), indicating a time-dependent cytostatic effect. Similarly, LiCl and MPA administered as single agents decreased BrdU incorporation, with values generally remaining above approximately 25–35%, indicating a weaker effect compared to combination treatments.

In contrast, combination treatments with imatinib plus LiCl or imatinib plus MPA resulted in the sustained suppression of BrdU incorporation across all examined time points (Figure 1A–C). By 96 h, BrdU incorporation in both combination groups declined to approximately 10–15%, which was lower than both control and imatinib monotherapy (Figure 1C,D), with significant differences observed between imatinib alone and combination treatments. These findings indicate that combining imatinib with LiCl or MPA enhances cytostatic efficacy, leading to the inhibition of DNA synthesis in 3D endometrial cancer spheroids.

### 3.2. Imatinib-Based Treatments Reshape Cell Cycle Dynamics in Spheroid-Derived Cells in a Time-Dependent Manner

**Figure 2:**
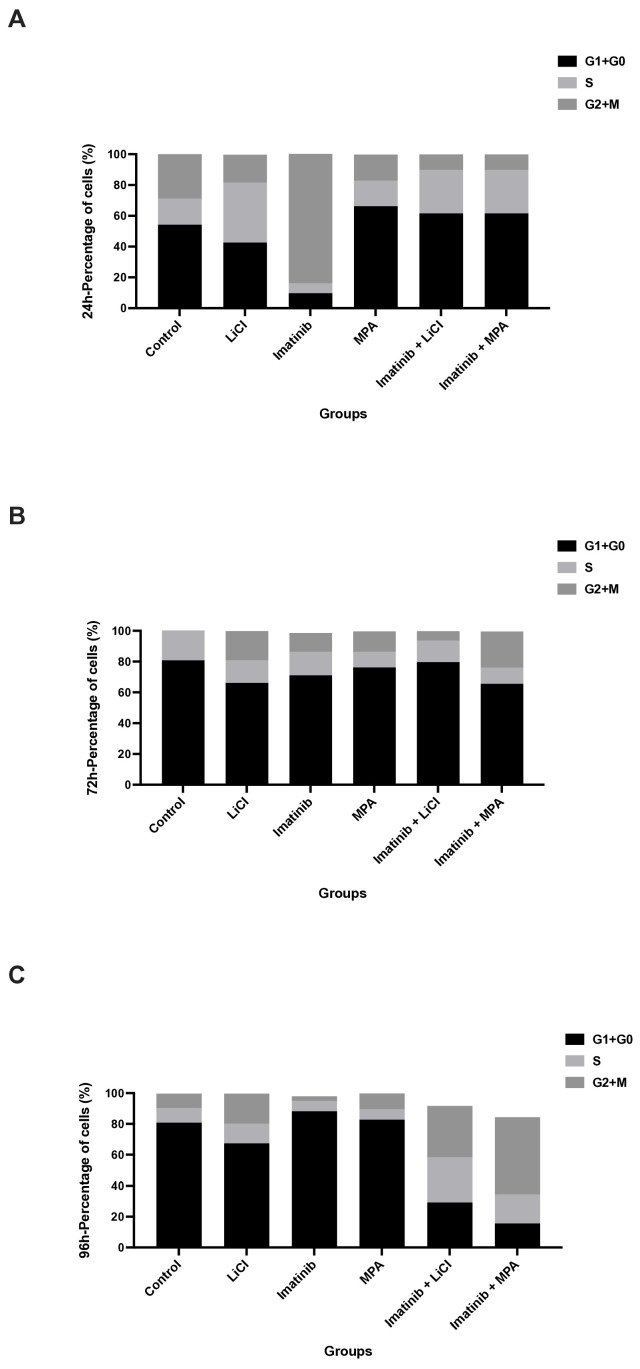
Imatinib-based treatments alter cell cycle distribution in 3D Ishikawa spheroids: (**A**–**C**) Distribution of cells across G_0_/G_1_, S, and G_2_/M phases at 24, 72, and 96 h following treatment. Stacked bar graphs represent the relative proportion of cells in each phase. A progressive increase in the G_0_/G_1_ fraction and a corresponding reduction in S-phase are evident, particularly in combination-treated groups. Quantitative analysis of cell cycle phases is provided in Figure 3.

To determine whether the observed reduction in proliferative activity was associated with altered cell cycle progression, DNA content analysis was performed at 24, 72, and 96 h following treatment (Figure 2A–C). Control spheroids exhibited a cell cycle distribution characterized by a substantial S-phase fraction, consistent with active proliferation.

Imatinib monotherapy induced a gradual redistribution of cells toward the G_0_/G_1_ phase, which became more apparent at later time points, reaching approximately 85–90% at 96 h. A similar temporal pattern was observed with LiCl treatment, with more evident G_0_/G_1_ accumulation at 96 h, whereas MPA promoted an earlier shift toward G_0_/G_1_ enrichment.

**Figure 3:**
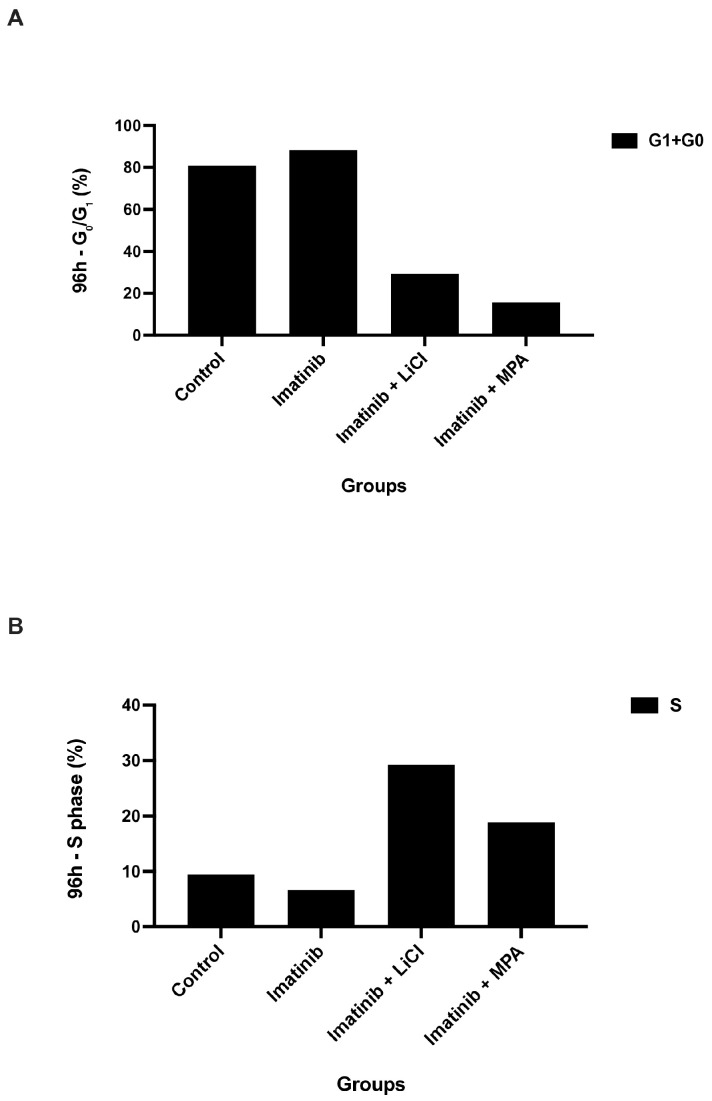
Descriptive quantification of cell cycle phase distribution in 3D Ishikawa spheroids: (**A**) Percentage of cells in the G_0_/G_1_ phase and (**B**) S phase at 96 h following treatment with imatinib and combination regimens. Values represent mean values from independent experiments and are presented for descriptive comparison. These data correspond to the cell cycle distributions presented in Figure 2.

Notably, combination treatments with imatinib plus LiCl or imatinib plus MPA resulted in a more pronounced accumulation of cells in the G_0_/G_1_ phase, accompanied by a reduction in S-phase fractions, particularly evident from 72 h onward (Figure 2B,C). By 96 h, S-phase fractions declined to approximately 10–20% in combination-treated groups, indicating a strong cytostatic response. Quantitative representation of these changes further supported the enrichment of G_0_/G_1_ populations and depletion of S-phase fractions in combination-treated groups relative to imatinib monotherapy (Figure 3).

Importantly, these alterations were not accompanied by consistent increases in the G_2_/M population, supporting the interpretation that growth suppression primarily reflects cell cycle arrest rather than mitotic blockade.

### 3.3. Imatinib-Based Combination Treatments Induce Potent G_0_/G_1_ Arrest and Suppress S-Phase Entry at 96 h

To further characterize the late-stage cytostatic response, the proportions of G_0_/G_1_- and S-phase cells were evaluated at 96 h (Figure 3A,B). Imatinib monotherapy increased the fraction of cells in the G_0_/G_1_ phase to approximately 85% and reduced S-phase entry to approximately 15% relative to control spheroids, consistent with a moderate cytostatic effect.

In contrast, both combination treatments further enhanced G_0_/G_1_ accumulation, approaching approximately 90%, and led to a more pronounced reduction in S-phase fractions to approximately 10% compared to imatinib alone. This effect was particularly evident in the imatinib plus MPA group, which showed the lowest proportion of S-phase cells among the treatment groups.

These findings indicate that imatinib-based combinations reinforce cell cycle arrest at later time points and support a sustained cytostatic response, consistent with the distribution patterns observed in Figure 2. Due to the descriptive nature of these measurements, formal statistical comparisons were not performed.

### 3.4. Annexin V/PI Analysis of Apoptosis in 3D Ishikawa Spheroids Following Treatment

**Figure 4:**
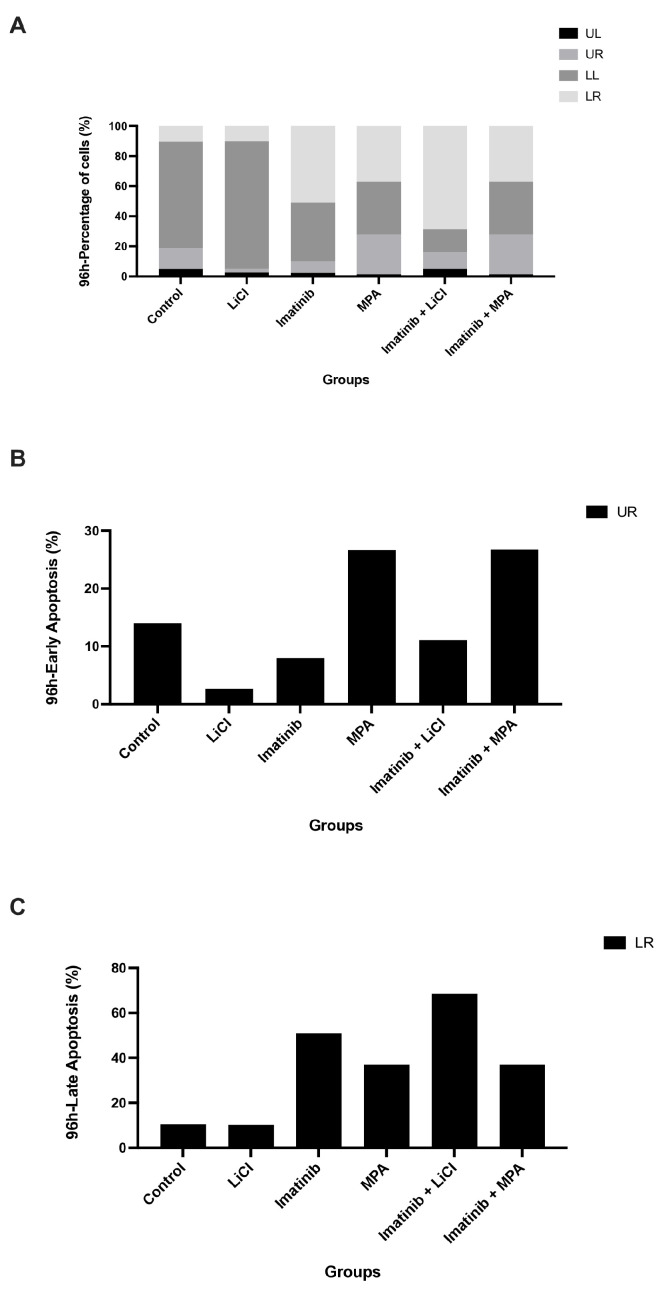
Imatinib-based treatments do not induce significant apoptosis in 3D Ishikawa spheroids: (**A**) Distribution of viable (UL), early apoptotic (UR), late apoptotic (LR), and necrotic (LL) cell populations following treatment. (**B**) Early apoptotic cells and (**C**) late apoptotic cells at 96 h. Values are presented for descriptive comparison. No marked increase in apoptotic fractions is observed in combination-treated groups compared to imatinib monotherapy.

To assess whether growth suppression was associated with cytotoxic effects, apoptotic and necrotic cell populations were analyzed at 96 h using Annexin V-FITC/PI staining (Figure 4A–C). Representative flow cytometry dot plots and the gating strategy are provided in Supplementary Figure S2. Control spheroids exhibited low baseline levels of apoptosis, with the majority of cells remaining in the viable (Annexin V^−^/PI^−^) fraction.

Neither imatinib monotherapy nor LiCl or MPA administered as single agents resulted in a substantial increase in apoptotic cell populations. Early apoptotic fractions generally remained within approximately 5–15% across most treatments, although MPA treatment showed a higher proportion of early apoptotic cells, reaching approximately 25–30% (Figure 4B). Similarly, late apoptotic (Annexin V^+^/PI^+^) fractions showed variability across treatments, ranging approximately from 10% to 70%, without a consistent pattern of apoptosis induction (Figure 4C).

Importantly, combination treatments with imatinib plus LiCl or imatinib plus MPA did not show a consistent increase in either early or late apoptotic populations relative to imatinib monotherapy (Figure 4B,C). Instead, apoptotic fractions remained variable and broadly comparable across treatment groups despite pronounced suppression of proliferation and cell cycle progression. These findings suggest that the growth-inhibitory effects of imatinib-based combinations in 3D spheroids are not primarily mediated by apoptosis but are consistent with a non-apoptotic cytostatic mechanism.

## 4. Discussion

In this study, we demonstrate that imatinib-based combination treatments suppress endometrial cancer cell growth predominantly through a non-apoptotic, cytostatic mechanism in a three-dimensional (3D) tumor model. Using Ishikawa endometrial carcinoma spheroids, we show that imatinib in combination with either lithium chloride (LiCl) or medroxyprogesterone acetate (MPA) induces a sustained reduction in DNA synthesis, as evidenced by a decrease in BrdU incorporation over time (Figure 1). This antiproliferative effect was accompanied by an accumulation of spheroid-derived cells in the G_0_/G_1_ phase and a corresponding depletion of S-phase populations (Figure 2 and Figure 3), while eliciting minimal apoptotic cell death (Figure 4). Together, these findings support the concept that durable control of pathological proliferation can be achieved through enforced cell cycle arrest within a 3D context rather than through induction of apoptosis [28]. Although BrdU penetration in 3D spheroids may be inherently limited compared to monolayer cultures, the consistent time-dependent trends observed across treatment groups support the reliability of the proliferation measurements.

Importantly, the magnitude of proliferation suppression observed in this study provides further insight into the biological nature of the response. BrdU incorporation declined to approximately 10–15% in combination-treated spheroids, compared to approximately 50–60% in imatinib monotherapy. This marked reduction indicates that the observed cytostatic effect is not merely a delay in cell cycle progression but reflects a substantial restriction of DNA synthesis capacity within the 3D tumor structure.

Our results extend previous observations indicating that imatinib monotherapy exhibits limited efficacy in endometrial cancer models, often producing only modest or transient antiproliferative effects [14]. In contrast, the addition of LiCl or MPA resulted in a markedly stronger and more persistent cytostatic response in 3D spheroids [26]. Importantly, time-course analyses extending to 96 h demonstrated that the growth-inhibitory state induced by combination treatments was not transient, as spheroid-derived cells remained largely excluded from S-phase at later time points (Figure 1). This distinguishes combination treatment from single-agent exposure and suggests that simultaneous modulation of multiple regulatory axes may be required to achieve stable proliferative arrest in a structured, tumor-like environment.

LiCl and MPA appear to contribute to this enhanced cytostatic effect through complementary but mechanistically distinct biological pathways. LiCl, a well-established inhibitor of glycogen synthase kinase-3β (GSK-3β), has been reported in various cancer models to induce cell cycle arrest and autophagy-associated responses without activating classical apoptotic pathways [25,29]. Consistent with previous studies in 3D endometrial cancer spheroids, LiCl alone exerted delayed yet detectable cytostatic effects, which were substantially reinforced when combined with imatinib. In contrast, MPA promoted a more rapid accumulation of spheroid-derived cells in the G_0_/G_1_ phase, in agreement with its established role in suppressing estrogen-driven proliferation and enforcing growth arrest in hormone-responsive endometrial carcinoma [30]. When combined with imatinib, MPA further intensified cell cycle blockade, likely reflecting convergence between hormonal and growth factor-dependent regulatory signaling. These effects were reflected in the cell cycle distributions observed in 3D spheroids, where combination treatments consistently increased the G_0_/G_1_ fraction relative to imatinib alone (Figure 2). Future studies should include the analysis of key cell cycle regulators such as cyclin D1, CDK4/6, p21, p27, and RB phosphorylation to further define the molecular basis of the observed cytostatic arrest. However, the expression or activation status of PDGFR or c-KIT was not directly assessed in this study, and therefore, the specific contribution of these targets remains to be clarified.

This interpretation is further supported by the quantitative cell cycle distribution. At 96 h, G_0_/G_1_ fractions approached approximately 90% in combination-treated groups, while S-phase populations declined to approximately 10–20%. Notably, the relative increase in G_0_/G_1_ was modest compared to imatinib alone, whereas the reduction in S-phase was more pronounced, suggesting that the primary effect of combination treatments is the restriction of S-phase entry rather than a simple accumulation of cells in G_0_/G_1_.

A key observation of this study is the dissociation between growth suppression and apoptosis in the 3D spheroid model. Despite pronounced inhibition of proliferation and a reduction in S-phase populations following combination treatment, Annexin V/PI analysis did not show a consistent increase in apoptotic cell death (Figure 4). This indicates that the dominant cellular response was cytostatic rather than cytotoxic. Such a response is particularly relevant in endometrial cancer, where resistance to apoptosis, often associated with TP53 alterations or dysregulation of intrinsic death pathways, limits the efficacy of conventional cytotoxic therapies [31]. By enforcing cell cycle exit without reliance on apoptotic machinery, imatinib-based combinations may therefore bypass resistance mechanisms that are accentuated in three-dimensional tumor architectures.

Consistent with this interpretation, apoptosis measurements revealed no coordinated increase in apoptotic populations across treatment groups, despite substantial suppression of proliferation. Early apoptotic fractions generally remained within a limited range, and late apoptotic fractions showed variability without a consistent pattern. Together, these findings reinforce the conclusion that growth suppression is primarily mediated through cytostatic mechanisms rather than the activation of cell death pathways.

Although molecular markers of senescence were not directly assessed in the present study, the sustained G_0_/G_1_ arrest observed in spheroid-derived cells is functionally consistent with a prolonged non-proliferative state, as supported by the quantitative cell cycle distribution shown in Figure 3, which has been associated with senescence-like phenotypes in previous reports [32]. Importantly, we deliberately refrain from definitively classifying this response as senescence, as canonical markers such as SA-β-galactosidase activity, p16^INK4a expression, or senescence-associated secretory phenotype (SASP) factors were not evaluated [33]. Nevertheless, the persistence of growth arrest over extended time points suggests that these treatments impose a stable restriction on tumor cell cycling within the 3D spheroid structure rather than a transient or readily reversible delay [34].

The broader concept of exploiting non-apoptotic growth arrest has gained increasing attention in cancer biology, particularly in experimental systems that recapitulate therapy resistance. Therapy-induced cytostasis in a 3D context may represent a biologically relevant endpoint, especially when considered alongside strategies aimed at eliminating arrested cells, such as immune-mediated clearance or senolytic approaches [28]. Although immune interactions were not addressed in the present study, prolonged arrest states have been proposed to modulate tumor–immune dynamics in vivo. Accordingly, our findings provide a rationale for future investigations exploring whether imatinib-based cytostatic strategies can be integrated with complementary therapeutic modalities. A schematic summary of the proposed cytostatic mechanism is presented in the graphical abstract.

Several limitations should be acknowledged. This study was conducted using a single endometrial cancer cell line in a three-dimensional spheroid model and focused primarily on functional outcomes rather than detailed molecular mechanisms. Future studies should evaluate whether similar cytostatic responses occur across additional endometrial cancer models, including hormone-resistant or high-grade subtypes, and should delineate the signaling pathways governing the observed cell cycle arrest. In addition, the reversibility of the observed growth arrest was not assessed, and drug withdrawal experiments will be necessary to determine whether this cytostatic state is transient or permanent. Furthermore, quantitative size standardization of spheroids and replicate-based statistical analyses were not fully implemented for all datasets, which may influence the interpretation of variability in cell cycle distributions. In vivo validation will also be required to determine whether growth suppression is maintained over longer periods and whether arrested tumor cells are ultimately eliminated or persist in a dormant state.

From a clinical perspective, sustained cytostatic enforcement may represent a complementary strategy in hormone-responsive or apoptosis-resistant endometrial cancer, particularly in settings where tumor burden control rather than rapid cytoreduction is the primary therapeutic objective. In advanced or recurrent disease, where repeated lines of cytotoxic chemotherapy often yield diminishing returns and increased toxicity, approaches that stabilize proliferative activity without triggering overt cell death may contribute to disease containment. While our findings remain preclinical and require validation in additional models and in vivo systems, they suggest that rational combinations of already clinically available agents may be repurposed to modulate tumor growth dynamics in a structured tumor context. The sustained cytostatic phenotype observed across multiple functional readouts (Figure 1, Figure 2, Figure 3 and Figure 4) supports the notion that coordinated targeting of proliferative signaling pathways may provide durable control of tumor growth. Such strategies may ultimately expand the therapeutic framework beyond apoptosis-dependent paradigms in selected endometrial cancer subtypes.

## 5. Conclusions

In conclusion, imatinib-based combination treatments with lithium chloride or medroxyprogesterone acetate suppress endometrial cancer cell growth in 3D Ishikawa spheroids by enforcing sustained G_0_/G_1_ cell cycle arrest without inducing prominent apoptotic cell death. These findings suggest that durable control of pathological proliferation can be achieved through non-apoptotic cytostatic mechanisms within a three-dimensional tumor context and underscore the relevance of 3D models in capturing growth-control responses that may not be fully reflected in conventional monolayer systems. Although preclinical, these results suggest that rational combinations of clinically available agents may provide a complementary strategy for disease stabilization in selected endometrial cancer settings, particularly in tumors exhibiting resistance to apoptosis-dependent therapies. Further translational and in vivo investigations will be required to determine their potential clinical applicability.

## Data Availability

The original contributions presented in the study are included in the article/Supplementary Material, further inquiries can be directed to the corresponding author.

## Supplementary Materials

The following supporting information can be downloaded at: https://www.mdpi.com/article/10.3390/biomedicines14040906/s1, Figure S1. Representative images of Ishikawa spheroids under control and treatment conditions: (**A**) Phase-contrast image of an untreated Ishikawa spheroid showing a compact structure. (**B**) Trypan Blue-stained image of spheroids following treatment, illustrating loss of membrane integrity and increased dye uptake. Images were acquired using an inverted microscope (Zeiss) at 40× magnification using two different imaging approaches (phase-contrast and Trypan Blue staining). Therefore, these images are presented for illustrative purposes and are not intended for direct morphological comparison. Scale bars = 100 µm. Figure S2. Representative Annexin V-FITC/PI flow cytometry dot plots showing apoptotic and necrotic cell populations in Ishikawa spheroids following treatment. Cells were stained with Annexin V-FITC and propidium iodide (PI) and analyzed by flow cytometry. Quadrant distribution indicates viable cells (Annexin V^−^/PI^−^, lower left), early apoptotic cells (Annexin V^+^/PI^−^, lower right), late apoptotic cells (Annexin V^+^/PI^+^, upper right), and necrotic cells (Annexin V^−^/PI^+^, upper left). Representative plots are shown for (**A**) control, (**B**) LiCl, (**C**) imatinib, (**D**) MPA, (**E**) imatinib + LiCl, and (**F**) imatinib + MPA groups. These plots illustrate the gating strategy and distribution of cell populations used for apoptosis analysis.

## Abbreviations

The following abbreviations are used in this manuscript:

3D	Three-Dimensional
BrdU	Bromodeoxyuridine
LiCl	Lithium Chloride
MPA	Medroxyprogesterone Acetate
PI	Propidium Iodide
PDGFR	Platelet-Derived Growth Factor Receptor
GSK-3β	Glycogen Synthase Kinase-3 Beta
DMSO	Dimethyl Sulfoxide
ER	Estrogen Receptor
