# Supplementary figures and images for: Targeting Apoptosis-Resistant Proliferation: Imatinib-Based Combinations Induce Durable Cytostatic Arrest in 3D Endometrial Cancer Spheroids
Source: Biomedicines. 2026 Apr 16;14(4):906. doi: 10.3390/biomedicines14040906 (PMC13112995; doi:10.3390/biomedicines14040906)

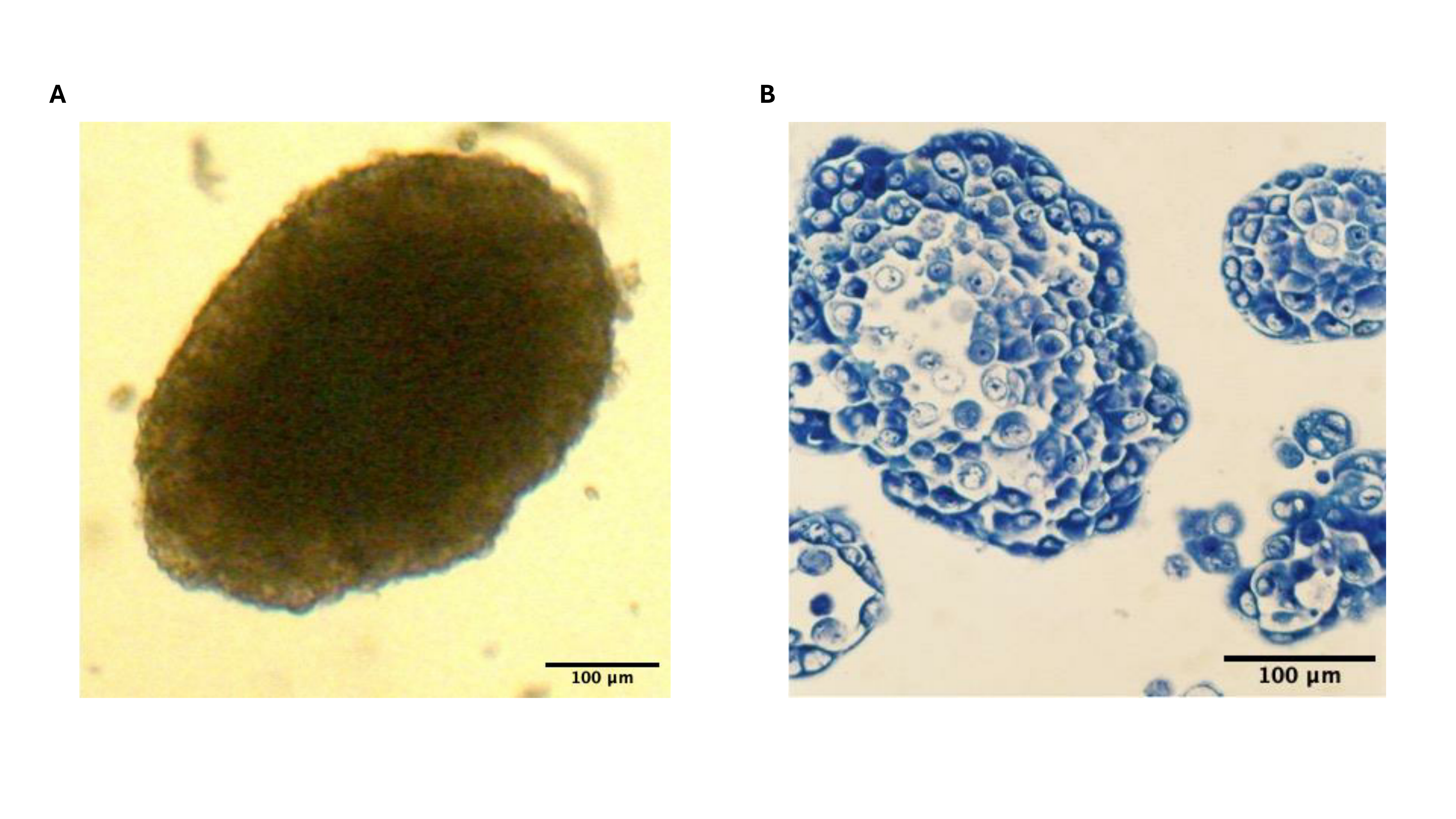

Supplement: Supplementary file 1 [file biomedicines-14-00906-s001.zip › supp figure 1.tif]

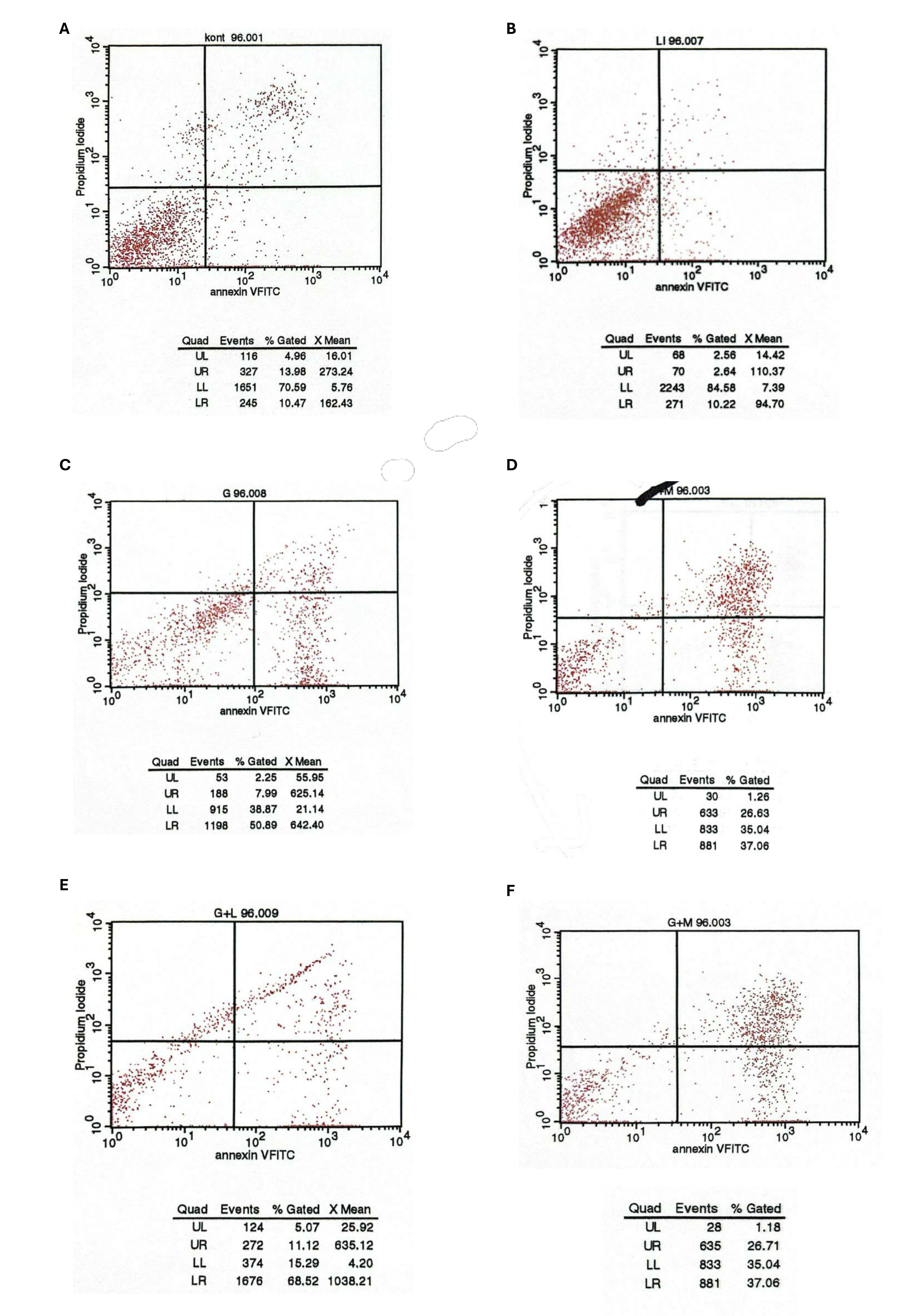

Supplement: Supplementary file 1 [file biomedicines-14-00906-s001.zip › Supp figure 2.tif]
